# Identifying patients at risk of prolonged hospital length of stay after total knee arthroplasty: A real-world study on the creation and validation of a cloud estimator

**DOI:** 10.17305/bb.2023.9156

**Published:** 2024-02-01

**Authors:** Song Chen, Minfei Qiang, Kunpeng Li, Xiong Wang, Wei Wang, Jun Xie

**Affiliations:** 1 Department of Orthopedics, The Quzhou Affiliated Hospital of Wenzhou Medical University, Zhejiang Province, China; 2 Department of Orthopedic Surgery, Zhongshan Hospital, Fudan University, Shanghai, China; 3 Department of Anesthesiology, The Second Affiliated Hospital of Nanjing Medical University, Nanjing, China; 4 Department of Orthopedics, Shanghai Baoshan Luodian Hospital, Shanghai, China

**Keywords:** Hospital length of stay (LOS), total knee arthroplasty (TKA), prediction, concordance statistic, calibration

## Abstract

Accurate prediction of the length of stay for patients undergoing total knee arthroplasty (TKA) is critical for efficient medical resource allocation. This study aimed to create a user-friendly model to assist this estimation process. A secondary analysis was conducted on 2676 patients who underwent elective primary TKA at a tertiary academic medical center in Singapore from January 2013 to June 2014. The eligible patients (*n* ═ 2600) were randomly divided into a training cohort (*n* ═ 2081) and a validation cohort (*n* ═ 519), at a ratio of 4:1. A prolonged hospital stay was defined as exceeding six days. Multivariable logistic regression was used to develop a prediction model, and an online calculator was created to facilitate its application. The model’s discrimination power, goodness-of-fit, and clinical applicability were evaluated. Additionally, models using other statistical methods were developed for performance comparison. The model includes predictors, such as age, operation duration, history of cerebrovascular accidents, creatinine levels, procedure site, the American Society of Anesthesiologists Physical status, hemoglobin levels, and primary anesthesia type. The model demonstrated robust discrimination power with a C statistic of 0.70 (95% confidence interval, 0.64–0.75), satisfactory goodness-of-fit (Hosmer–Lemeshow test, *P* ═ 0.286) and was applicable when thresholds were between 0.08 and 0.52, based on decision curve analysis. A predictive model was developed that can be used to identify patients who are likely to require an extended stay following TKA. This could assist in planning bed availability and guiding therapeutic decisions.

## Introduction

Total knee arthroplasty (TKA) has emerged as a highly effective treatment for end-stage knee osteoarthritis, significantly improving the quality of life for patients worldwide [1, 2]. In line with this progress, enhanced recovery after surgery (ERAS) programs have been developed and advocated, with the aim of improving postoperative functional recovery and optimize the utilization of hospital resources [3, 4]. A key measure of this efficiency is the length of stay (LOS) in the hospital following surgery.

Several studies have been conducted globally to understand and manage LOS for TKA. The typical hospital stay after TKA varies across different regions. In the USA, it is around three to five days, whereas, in European countries, the average LOS is reported to be five to seven days [5–8]. In Asia, countries like China and Singapore report an average LOS of approximately four to five days [9, 10]. However, these are average lengths and individual cases may significantly deviate from these norms. An extended hospital stay, usually defined as a stay beyond six days, has implications for patient health and well-being, but also for hospital resource allocation.

Efficient hospital management necessitates accurate prediction and management of the LOS. Discrepancies between the actual and predicted LOS can lead to either overcrowding or underutilization, both of which present challenges for patient safety and hospital finances [11–13]. Thus, preventing unnecessary extensions of patients’ hospital stays is a critical objective. Despite the use of existing prognostic factors to anticipate extended hospitalization following TKA, such as age, comorbidities, and ASA score—these parameters exhibit notable limitations. They often lack specificity, fail to encapsulate all relevant clinical variables, and varying predictive accuracy across different studies and environments [6, 9, 10]. This underscores the need to develop reliable predictive models for LOS. Such models can aid in efficient resource allocation, enable surgeons to devise comprehensive preoperative plans, and assist patients and their families in planning for work absences or post-hospital care.

Several predictive models have been proposed in the past, most commonly using logistic regression approaches to provide personalized, evidence-based risk estimates [14–16]. Advances in digital health technology have further led to the development of user-friendly tools, such as nomograms and online calculators, enhancing patient care decision making. This study builds upon these efforts, aiming to develop and validate a precise, individualized prediction model for LOS following TKA. This model, integrated into a user-friendly web-based calculator, incorporates readily accessible clinical predictors to provide rapid and accurate predictions.

## Material and methods

### Data source

The datasets employed in this research are available from the Dryad digital repository [https://datadryad.org/stash/dataset/doi:10.5061%2Fdryad.73250]. Dryad is an open-source database that offers a wide array of discoverable, freely reusable, and citation-worthy research data. All private information within the database has been anonymized. 

### Study design and participants

The present analysis was a secondary examination based on a retrospective cohort research [10], which included 2676 patients treated with TKA at a tertiary academic medical center in Singapore between January 2013 and June 2014. We excluded 54 patients lacking essential information. Of the remaining 2622 patients, we excluded 22 who had undergone revisions. To avoid selection bias and optimize data utilization, we included almost all predictive variables provided by Abdullah et al. for analysis [10]. These variables are summarized in Table S2. Relevant definitions were elucidated at length in the original article [10]. The outcome under consideration was an extended LOS, defined as more than six days from admission to discharge. This benchmark represents the median LOS for the entire sample and aligns with previous studies [10, 17, 18].

### Missing data

To enhance statistical robustness and reduce bias, missing data among eligible patients was imputed using *k*-nearest neighbor (KNN) imputation with *k* set to 10. Subsequently, the obtained imputation data were randomly stratified into two subsets (i.e., training and validation cohorts) at a 4:1 ratio. These subsets were then compared with the complete dataset (i.e., data with all missing values removed) and no significant differences were discerned. Further details of the statistical analysis are provided in the appendix.

### Sample size calculation

The R package *“pmsampsize”*, version 1.1.2 (https://cran.r-project.org/web/packages/pmsampsize), was used to compute the required minimum sample size for training. To construct a multivariable prediction model for the binary outcome, 16 candidate predictor parameters were selected. Additionally, based on previous evidence [10], we anticipated the outcome prevalence to be 0.192, and projected the R-squared value of the new model to have a lower bound of 0.12. Utilizing PASS 15 (NCSS, LLC., Kaysville, UT, USA), a power calculation was performed on the validation sample size. The area under the receiver operating characteristic (ROC) curve (AUC; comparable to the concordance statistic [C statistic]) was expected to be at least 0.80. A two-tailed test with an alpha error of 0.05, beta error of 0.1, and a power of 0.90 was adopted. Consequently, the minimum sample size required for the training cohort was 1119 patients with 216 instances, while the validation cohort requires 45 patients with 12 events. The eligible population is adequate for both model construction and validation.

### Ethical statement

All analyzed data is from the research conducted by Abdullah et al. [10]. In compliance with the Helsinki Declaration, local ethical clearance for data collection was granted. Institutional Review Board (IRB) approval was obtained (Singhealth CIRB 2014/651/D), and the requirement for written informed consent was waived.

### Statistical analysis

Continuous variables were reported as medians with interquartile ranges (IQRs) and compared using an unpaired Mann–Whitney test. Categorical variables were compared using the χ^2^ test. For each significant continuous variable within the training cohort, we used a restricted cubic spline (RCS) with five knots at the 5th, 35th, 50th, 65th, and 95th percentiles to flexibly model its association with a prolonged LOS. The RCS model was adjusted for variables, including age, race, patient comorbidities, creatinine, American Society of Anesthesiologist (ASA) status, type of anesthesia, procedure site, and operation duration (OD). To relax the assumptions of relationship, identified nonlinear continuous predictors were further classified according to corresponding reference points indicated by RCSs and horizontal lines with an odds ratio of one. Then, linear continuous and acquired categorical predictors were evaluated using univariate logistic regression analysis to determine the independent risk factors for a longer LOS. All variables significantly associated with an extended LOS were suitable for the backward stepwise multivariate analysis. Subsequently, the “*rms*” package was used to develop a nomogram based on the findings of a multivariate logistic regression analysis. The nomogram relied on the proportional conversion of each regression coefficient in multivariate logistic regression to a 0- to 100-point scale, assigning 100 points to the variable with the highest β coefficient (absolute value) to denote its effect. Total points were generated by accumulating points across independent variables, which were then translated into projected probabilities. The predictive capacity of the nomogram was assessed using the C statistic [19] and calibrated using 1000 bootstrap samples to mitigate overfit bias. Further examination of the calibration was conducted using the Hosmer–Lemeshow goodness-of-fit test with deciles of estimated risk. Additionally, we calculated the variance inflation factor (VIF) to examine the collinearity of each predictor in the prediction model and conducted a formal sensitivity analysis, as described by VanderWeele and Ding [20], to account for the potential impact of unmeasured predictors on the procured estimate.

Based on the nomogram, the total scores of each patient were determined for the clinical application of the model. The ideal cutoff values were obtained by optimizing the Youden index (i.e., sensitivity + specificity − 1). The sensitivity, specificity, predictive values, and likelihood ratios were used to evaluate the precision of the appropriate cutoff value. To assess the model’s clinical utility, decision curve analysis (DCA) was included as a supplement.

In addition, we attempted alternative methods of modeling to verify the robustness of the model. One such approach was to use the best subsets regression (BSR) with the Bayesian information criterion [21] to filter predictors and construct a new model. The least absolute shrinkage and selection operator (LASSO) [22] offered another method of repeating this process. Subsequently, the Delong’s test was used on the validation set to compare the AUCs of these models.

Finally, considering LOS as a discrete variable, we established the fourth model with a quasi-Poisson regression. Due to overdispersion for model fit, we abandoned the standard Poisson method. Likewise, we implemented variable elimination using the backward stepwise regression method. The root mean square error (RMSE) of the training and validation sets was used to assess the model’s performance.

All statistical analyses were performed using the R programming language. The additional R packages used in this work were: “*pROC*”, “*car*”, “*caret*”, “*splines*”, “*EValue*”, “*rmda*”, “*ggDCA*”, “*leaps*”, “*glmnet*”, “*qcc*”, and “*ggplot2*”. Except for the pairwise comparison of AUCs, statistical significance levels were established using two-sided tests, and *P* < 0.05 was considered statistically significant. In this instance, we applied the Bonferroni correction, and tests with a limit of 0.017 were used to adjust the *P* values.

## Results

### Baseline characteristics

A total of 2600 adult patients who underwent TKA were included in the design dataset. The KNN imputation was used to account for missing data for race in four (0.2%), body mass index (BMI) in 66 (2.5%), and creatinine in 292 (11.2%) cases. The median patient age was 66 years (IQR, 61–72) years, with 1977 (76.0%) patients being female, 2192 (84.3%) being Chinese, and 547 (21.0%) experiencing a prolonged LOS. A similar population distribution was detected in the complete data, as shown in Table S1. No statistical differences emerged in comparisons between the imputation and complete data sets (all *P* > 0.05).

Of the 2600 patients, 2081 and 519 were allocated to the training and validation cohorts, respectively. No statistical differences were observed in the baseline characteristics between the two cohorts (all *P* > 0.05). The median hospital LOS was four days (IQR, 3–6) and the prolonged LOS rate was 21.0% in both cohorts (Table S2).

In the training cohort, patients with extended hospital stays demonstrated a higher median age (66 vs 68 years, *P* < 0.001), lower median hemoglobin (Hb) concentration (13.2 vs 12.8 g/dL, *P* < 0.001), longer OD (80 vs 85 min, *P* < 0.001), and a higher incidence of diabetes mellitus (DM, *P* ═ 0.026), ischemic heart disease (IHD, *P* ═ 0.011), and previous cerebrovascular accidents (CVA; *P* < 0.001). Furthermore, patients with creatinine levels exceeding 2 mg/dL (*P* ═ 0.002), higher ASA status (*P* < 0.001), those receiving general anesthesia (*P* ═ 0.002), and those undergoing bilateral TKA (*P* < 0.001) were identified as experiencing prolonged hospital stays. No statistical differences were noted between these two groups in terms of sex or BMI (Table S3).

### Model specifications and predictors

As shown in Figure S1, the continuous variable Hb did not satisfy the requirements for a linear relationship (*P*_non-linear_ < 0.05). We transformed it into a categorical variable using reference points as cut-off values for the subsequent univariable logistic analysis. Significant differences were found between patients with normal and extended LOS in this converted variable (Table S3).

The results of the univariate logistic analysis are presented in Table 1. Backward stepwise selection using Akaike’s Information Criterion in the logistic regression modeling identified the following 10 predictors that had the strongest association with prolonged LOS: Age, race, DM, CVA, Hb, creatinine, ASA status, anesthesia types, procedure site, and OD. On multivariable analysis, each of the following was independently associated with the risk of prolonged LOS: Age (odds ratio [OR], 1.04; 95% confidence interval [CI], 1.03–1.06; *P* < 0.001), CVA (OR 2.94; 95% CI 1.49–5.77; *P* ═ 0.002), Hb at 13 g/dL or above (OR 0.68; 95% CI 0.54–0.85; *P* < 0.001), creatinine above 2 mg/dL (OR 3.17; 95% CI 1.19–8.47; *P* ═ 0.021), ASA level of 3 (OR 2.04; 95% CI 1.36–3.07; *P* < 0.001), regional anesthesia (OR 0.69; 95% CI 0.55–0.87; *P* ═ 0.002), bilateral procedure sites (OR 2.93; 95% CI 1.94–4.22; *P* ═ 0.002), and OD (OR 1.01; 95% CI 1.00–1.01; *P* ═ 0.006) (Table 1).

**Table 1:** Logistic regression analysis of LOS for patients after TKA in the training cohort

**Variables**	**Univariable**		**Multivariable**	
	**OR (95% CI)**	***P* value**	**aOR (95% CI)**	***P* value**
*Factors Selected by Stepwise Analysis*				
Age, years	1.04 (1.02, 1.05)	**<0.001**	1.04 (1.03, 1.06)	**<0.001**
**Race**				
Chinese	1 [Reference]	NA	1 [Reference]	NA
Malay	0.59 (0.35, 0.93)	**0.023**	0.64 (0.38, 1.08)	0.093
Indian	1.08 (0.68, 1.66)	0.736	1.17 (0.73, 1.87)	0.509
Others	1.63 (0.90, 2.84)	0.101	1.70 (0.95, 3.07)	0.078
**DM**				
No	1 [Reference]	NA	1 [Reference]	NA
Yes	1.35 (1.03, 1.75)	**0.029**	1.29 (0.98, 1.71)	0.069
**CVA**				
No	1 [Reference]	NA	1 [Reference]	NA
Yes	3.12 (1.61, 5.99)	**0.001**	2.94 (1.49, 5.77)	**0.002**
**Hb, g/dL**				
<13	1 [Reference]	NA	1 [Reference]	NA
≥13	0.62 (0.50, 0.77)	**<0.001**	0.68 (0.54, 0.85)	**<0.001**
**Creatinine, mg/dL**				
≤2	1 [Reference]	NA	1 [Reference]	NA
>2	4.23 (1.68, 10.85)	**0.003**	3.17 (1.19, 8.47)	**0.021**
**ASA status**				
1 or 2 3	1 [Reference] 2.54 (1.73, 3.70)	NA **<0.001**	1 [Reference] 2.04 (1.36, 3.07)	NA **<0.001**
**Type of anesthesia**				
General	1 [Reference]	NA	1 [Reference]	NA
Regional	0.71 (0.57, 0.88)	**0.002**	0.69 (0.55, 0.87)	**0.002**
**Procedure site**				
Unilateral	1 [Reference]	NA	1 [Reference]	NA
Bilateral	3.21 (2.30, 4.46)	**<0.001**	2.93 (1.94, 4.42)	**<0.001**
OD, mins	1.01 (1.01, 1.02)	**<0.001**	1.01 (1.00, 1.01)	**0.006**
*Factors Not Selected by Stepwise Analysis*				
**IHD**				
No	1 [Reference]	NA	NA	NA
Yes	1.72 (1.12, 2.59)	**0.015**		

Bold values indicate statistical significance. LOS: Length of stay; TKA: Total knee arthroplasty; DM: Diabetes mellitus; CVA: Cerebrovascular accident; Hb: Haemoglobin; ASA: American Society of Anesthesiologist; OD: Operation duration; IHD: Ischaemic heart disease; OR: Odds ratio; aOR: Adjusted odds ratio; CI: Confidence interval; NA: Not applicable.

We also created additional models using BSR and LASSO approaches. Figure S2 illustrates the processes of variable selection, and the final selection of predictors is presented in Table S4 . As shown, the LASSO retains the fewest variables, followed by the BSR. Interestingly, variables selected by these three different methods show a layer-upon-layer containment. Age, CVA, Hb, ASA status, procedure site, and OD are common predictors chosen by the mentioned methodologies. In the validation set, models based on these selected predictors demonstrated comparable discriminatory power. The AUCs for the original model, BSR, and LASSO were 0.70 (95% CI 0.64–0.75), 0.69 (95% CI 0.64–0.75), and 0.69 (95% CI 0.63–0.74), respectively. Compared with the AUC of the original model, there were no differences for BSR (*P* ═ 0.781) and LASSO (*P* ═ 0.403).

Finally, the fourth model was established using the quasi-Poisson regression after treating the LOS as a discrete variable. The variables included are listed in Table S5. Similar results appeared again in the multivariable analysis, with age, Hb, creatinine, ASA status, type of anesthesia, procedure site, and OD in addition to CVA that were identified as independent factors for extended LOS. The RMSEs of this model were 6.28 and 5.43 days when applied to the training and validation cohorts, respectively. Therefore, we believe that CVA might be crucial in predicting the prolonged LOS, given its appearance in the previous three models, and that the original model was comprehensive as it included all predictors from other models.

### Model development and validation

**Figure 1.**
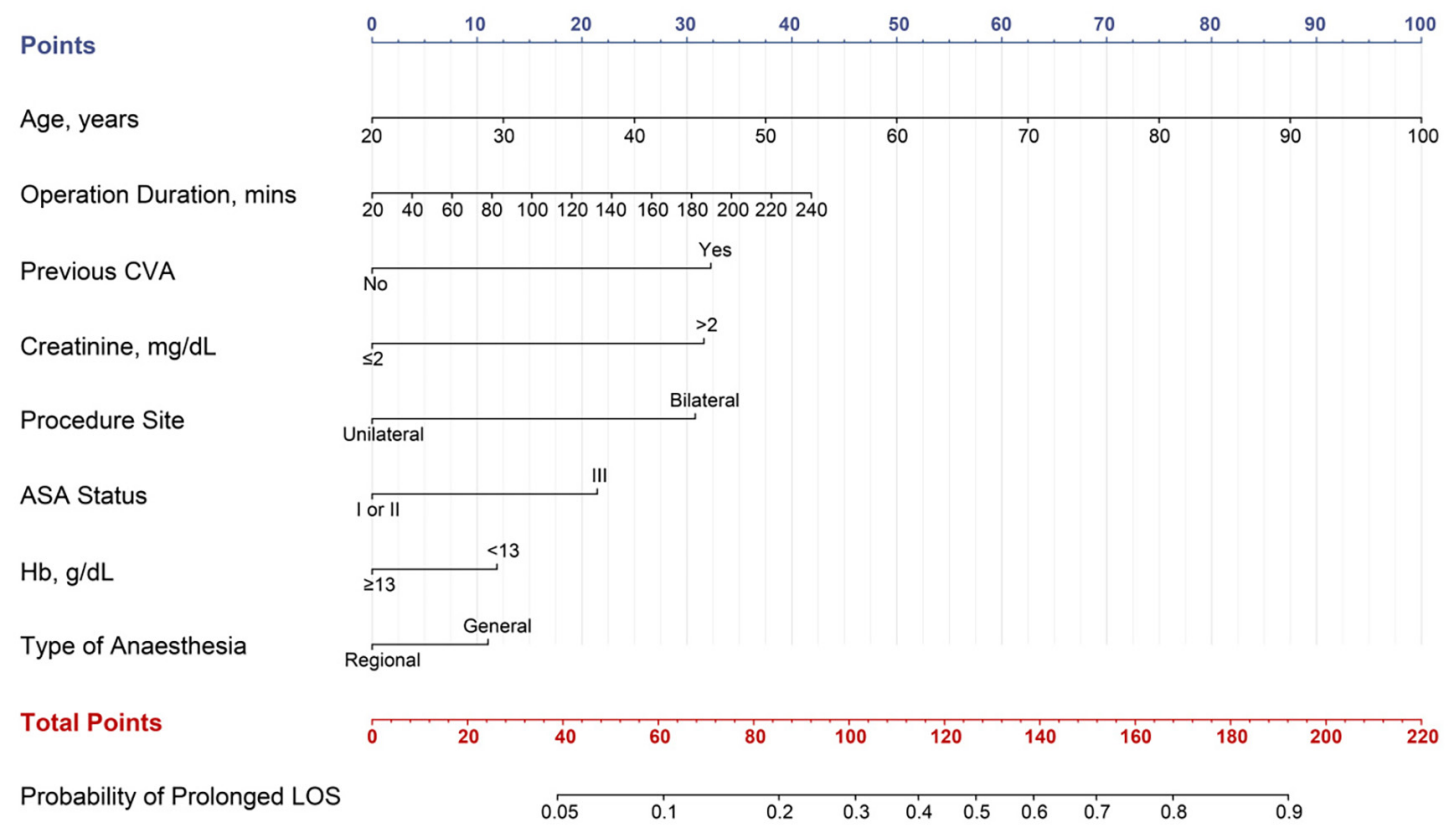
**Nomogram to estimate the risk of prolonged LOS.** Using a nomogram, we first determined the location of the axis corresponding to each variable and drew a vertical line to the “Points” axis to obtain a score, then summed scores from all variables and drew a second vertical line from the “Total Points” axis to the “Probability of Risk” axis to calculate the predicted probability. LOS: Length of stay; CVA: Cerebrovascular accident; ASA: American Society of Anesthesiologist; Hb: Hemoglobin.

Using identified independently related risk factors from the original model, a nomogram was created to estimate the risk of prolonged LOS (Figure 1). VIFs of 1.45 or below for predictors indicate the absence of collinearity. We also computed the E-value, a conventional method for quantifying the potential impact of unmeasured predictors on the achieved estimations, and reported it in Figure S3 for each predictor. The lowest value for OD was 1.06, suggesting limited robustness to unmeasured confounders. However, other predictors showed greater resistance to unmeasured confounders, except for a significant unmeasured confounder that was significantly associated with extended LOS. To facilitate the model’s usage in clinical settings, we developed a web-based calculator (https://songandwen.shinyapps.io/PredictLOS) (Figure S4).

**Figure 2.**
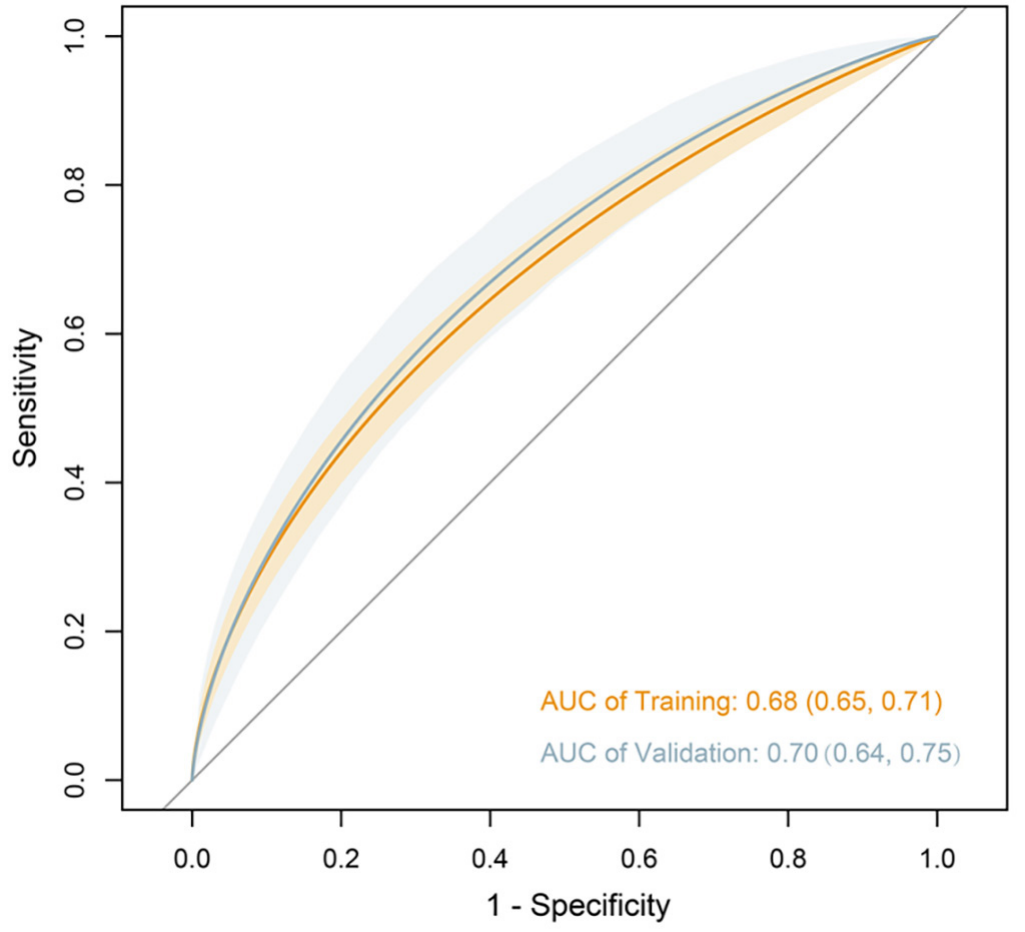
**Receiver operating characteristic curves for validating the discrimination of the model.** AUC: Area under the curve.

**Figure 3.**
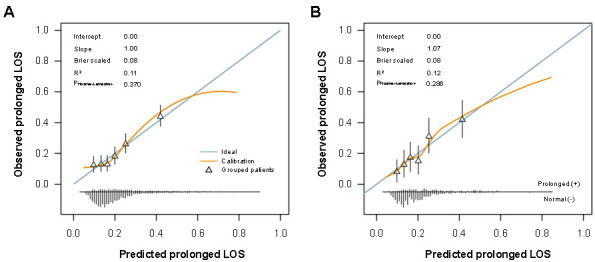
**Calibration plots for a model to predict hospital LOS in TKA patients.** (A) In the training cohort (*n* ═ 2081); (B) In the validation cohort (*n* ═ 519). TKA: Total knee arthroplasty; LOS: Length of stay.

In terms of model performance testing, our initial step was internal validation, which we performed using the bootstrap validation method. The model showed a certain discrimination capability in estimating prolonged LOS with an unadjusted C statistic of 0.68 (95% CI, 0.65–0.71) and a bootstrap-corrected C statistic of 0.68 (Figure 2). Furthermore, calibration plots showed excellent agreement between risk estimation and actual prolonged LOS (Figure 3A; *P* ═ 0.370). In the validation cohort, the model showed a marginally higher C statistic of 0.70 (95% CI, 0.64–0.75) for predicting prolonged LOS (Figure 2). In addition, the risk estimation yielded a respectable calibration curve (Figure 3B; *P* ═ 0.286).

### Clinical usage of model

We hypothesized that nomogram scores exceeding a defined threshold would indicate a prolonged hospital LOS for a patient, while scores below the threshold would suggest otherwise. As a result, we calculated a total score for each patient and determined the optimal cutoff value to be 115. The sensitivity, specificity, positive predictive value (PPV), and negative predictive value (NPV) were 56.8%, 73.7%, 36.6%, and 86.5% in the training cohort, and 56.9%, 72.0%, 35.0%, and 86.2% in the validation cohort, respectively (Table 2).

**Table 2:** Performance metrics for estimating the risk of prolonged LOS for patients after TKA

**Performance metrics**	**Value (95% CI)**	
	**Training set**	**Validation set**
Cutoff score*	115	115
Sensitivity, %	56.8 (52.1, 61.5)	56.9 (47.1, 66.2)
Specificity, %	73.7 (71.5, 75.8)	72.0 (69.3, 76.2)
Positive predictive value, %	36.6 (34.0, 41.2)	35.0 (28.1, 42.6)
Negative predictive value, %	86.5 (84.1, 87.8)	86.3 (82.0, 89.6)
Positive likelihood ratio	2.16 (1.93, 2.43)	2.03 (1.62, 2.54)
Negative likelihood ratio	0.59 (0.52, 0.65)	0.60 (0.48, 0.75)

*Optimal cutoff scores were determined by maximizing the Youden index (i.e., sensitivity + specificity − 1). CI: Confidence interval; LOS: Length of stay; TKA: Total knee arthroplasty.

**Figure 4.**
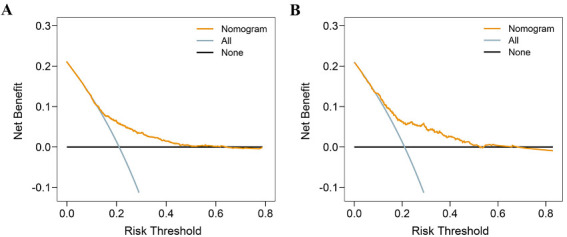
**Decision curve analysis for the model predicting hospital LOS in TKA patients.** The black line represents a scheme to make all patients have a normal LOS. The light blue line represents a protocol that led to the extended hospital LOS of all patients. The yellow line represents the net benefits of the clinical application of the model. (A) In the training cohort (*n* ═ 2081), the model is applicable when thresholds are in the range between 0.11 and 0.61, due to the net benefits being greater than zero; (B) In the validation cohort (*n* ═ 519), the valid range is between 0.08 and 0.52. TKA: Total knee arthroplasty; LOS: Length of stay.

In addition, we utilized DCAs to assess the net benefit of the model for decision making. As illustrated in Figure 4, the model was applicable in the training cohort when thresholds fall between 0.11 and 0.61, as net benefits exceed zero. In the validation cohort, the valid range was between 0.08 and 0.52.

## Discussion

This study presents a unique model for predicting hospital stay length following TKA based on a dataset from a single center in Singapore. Our model, which included predictors, such as age, CVA, Hb, creatinine, ASA status, type of anesthesia, procedure site, and OD, emerged as a tool with superior discriminatory capability compared to both BSR and LASSO models. The introduction of predictors that can be determined before or immediately after surgery enables an individual to forecast at an early stage and the development of an initial hospitalization protocol. This is a unique aspect of our model and an advancement from current prediction tools that often require more time and information for a valid prediction.

The current research emphasized the modifiable predictors—Hb levels, type of anesthesia, and OD. The existing literature confirms the importance of these factors, and our analysis further substantiates their pivotal role in the duration of hospital stay following TKA. We used the RCS method to ascertain a threshold value of 13 g/dL. This figure aligns with the recent suggestion to define preoperative anemia using a gender-specific cut-off of 13.0 g/dL. We discerned that the likelihood of an extended LOS increased by 32% for anemic patients compared to non-anemic patients after adjustment for covariates. This figure closely mirrors the findings of Montserrat et al., whose investigation identified preoperative anemia as an independent factor correlating with prolonged hospital stay (adjusted OR 1.31; 95% CI 1.11–1.54; *P* ═ 0.002) [23]. An observational study conducted by Zaninetti et al. also reported a significant association between the presence and degree of anemia and hospital stay length [24]. These authors underscore the need for prompt identification of preoperative anemia to diagnose and promptly address any potentially reversible causes.

In the context of anesthesia type, a meta-analysis conducted by Johnson et al. [25] suggested that neuraxial anesthesia significantly reduces the length of hospital stay for patients undergoing total hip or knee arthroplasty compared to general anesthesia. Similarly, Nishi et al. [26] compared various regional and general anesthesia in elderly patients post-hip fracture surgery and concluded that the former was more effective in shortening the hospital stay duration. More recently, Alexander et al. [27] used a comprehensive, verified dataset along with a variety of statistical methodologies to mitigate confounding variables, thereby revealing a correlation between reduced hospital stay length and the use of regional anesthesia in ankle surgery patients. In our research, the risk of extended hospital stays for patients subjected to regional anesthesia was approximately two-thirds that of individuals who underwent general anesthesia. Unfortunately, this additional analysis could not step into detailed comparisons among distinct subgroups of regional anesthesia due to the limitations of the original dataset. We aim to address these issues in a future prospective study, using our own case histories.

In addition, the OD was considered as a continuous variable and evaluated using both logistic and quasi-Poisson regression methods, despite the odds ratio (or risk ratio [RR]) values were close to one. This was expected given that OD was measured in minutes. When examining similar relationships, Allan et al. [28] found similar results (OR ═ 1.001). Therefore, it becomes essential to determine ways to safely reduce the duration of the procedure. Recently, innovative and clinically applicable preoperative planning strategies have emerged, such as those utilizing computer assistance or 3D printing, each reported to significantly decrease operative times and reduce postoperative complication rates [29–31]. This could potentially provide surgeons a practical solution to the aforementioned issue.

Age, CVA, ASA status, and procedure site were non-modifiable predictors in our study. We found that the risk of prolonged hospitalization increased by 0.04 times for each advancing year of age, a finding that aligns with other research [10, 32]. This could be partially attributed to the common complications associated with aging, which include cardiovascular disease, hypertension, type 2 diabetes, and chronic obstructive pulmonary disease [33]. Such complications categorize patients as high-risk, potentially requiring additional treatment and thereby extending their hospital stay. We also observed that patients with a prior CVA had a significantly longer LOS. Specogna et al. noted that patient multimorbidity, especially hypertension, was a strong predictor of extended hospitalization and increasing costs following spontaneous intracerebral hemorrhage [34]. Moreover, several studies [34, 35] have suggested that elevated ASA scores correlate with a prolonged hospital stay for TKA patients. We concur with this view. Our analysis confirmed that patients with an ASA score of three had approximately twice the risk of an extended stay compared to those with a score of one or two. These patients require especially careful management.

In addition, the logistic regression approach has been chosen to construct the predictive model, which might be limited by its linearity assumption. Although considerable effort was made to build RCS models to examine this assumption, complex relationships between residual predictor and response variables may still be overlooked. These challenges can be easily addressed by machine learning algorithms that do not require strict data structure assumptions and are capable of learning complex functional forms using non-parametric methods [36]. In a study based on deep learning algorithms to predict the number hospital stay days for patients with primary TKA, the model built by Ramkumar et al. had an AUC value of 0.74 [37], which was marginally better than our model. However, their model incorporated 16 predictor variables, twice the number of variables in our model, and such a complex model is not conducive to routine clinical application. Moreover, due to the “black box” phenomenon [38, 39], the specific influence of each predictor on the outcome is obscured, making it harder to translate the findings of machine learning models into actionable insights for patient care. In our model, the influence of each predictor on the LOS can be explained by the associated coefficient, which indicates the change in odds for a one-unit increase in the predictor, holding other variables constant. Therefore, despite the marginally lower AUC, our logistic regression model can provide more reliable and interpretable predictions with fewer variables.

Our study has several limitations. First, there was missing data for race, BMI, and creatinine. In this instance, it was assumed that the missing data occurred at random, hence KNN imputation was utilized to minimize selection bias. Furthermore, we found no significant statistical difference between the complete and the imputed data. Second, as this is a secondary analysis using fixed data, we may have overlooked significant predictors, such as preoperative motivation and living status. Therefore, we quantified the unmeasured confounders to test the robustness of our model. Third, all analyses were based on the data from a single institution; it is necessary to validate the results from other centers. A prospective study is also required to further confirm the reliability of the model. In light of this, the web calculator was created to facilitate the implementation of these requirements. Fourth, we acknowledge that the type of prostheses used in the TKA surgeries, ranging from modern KR implants to classic CR implants, may influence the recovery times and patient outcomes [40]. Future studies may consider investigating the effects of different types of prostheses on hospital stay duration. Lastly, while the model was able to predict extended LOS with acceptable accuracy, there is still room for improvement, especially when important clinical decisions need to be made.

## Conclusion

We developed a predictive model that can be used to identify patients likely to require an extended stay following TKA. This could assist in planning bed availability and guiding therapeutic decisions.

## Supplemental Data

Supplementary data are available at the following link: https://www.bjbms.org/ojs/index.php/bjbms/article/view/9156/2881
